# Association of Vital Pulp Therapy Outcomes with Tooth Type, Arch Location, Treatment Type, and Number of Surfaces Destroyed in Deciduous Teeth: A Retrospective Study

**DOI:** 10.3390/ijerph18157970

**Published:** 2021-07-28

**Authors:** Manahil Maqbool, Tahir Yusuf Noorani, Norsamsu Arni Samsudin, Mohamad Arif Awang Nawi, Giampiero Rossi-Fedele, Mohmed Isaqali Karobari, Pietro Messina, Giuseppe Alessandro Scardina

**Affiliations:** 1 Paediatric Dentistry Unit, School of Dental Sciences, Universiti Sains Malaysia, Health Campus, Kota Bharu 16150, Kelantan, Malaysia; manahil.maqbool@gmail.com (M.M.); arnisamsudin@usm.my (N.A.S.); 2 Conservative Dentistry Unit, School of Dental Sciences, Universiti Sains Malaysia, Health Campus, Kota Bharu 16150, Kelantan, Malaysia; dr.isaq@gmail.com; 3 Conservative Dentistry Unit, Hospital Universiti Sains Malaysia, Health Campus, Kota Bharu 16150, Kelantan, Malaysia; 4 Paediatric Dentistry Unit, Hospital Universiti Sains Malaysia, Health Campus, Kota Bharu 16150, Kelantan, Malaysia; 5 Biostatistics Unit, School of Dental Sciences, Universiti Sains Malaysia, Health Campus, Kota Bharu 16150, Kelantan, Malaysia; mohamadarif@usm.my; 6 Adelaide Dental School, University of Adelaide, Adelaide 5005, Australia; giampiero.rossi-fedele@adelaide.edu.au; 7 Department of Conservative Dentistry & Endodontics, Saveetha Dental College & Hospitals, Saveetha Institute of Medical and Technical Sciences University, Chennai 600077, Tamil Nadu, India; 8 Department of Surgical, Oncological and Stomatological Disciplines, University of Palermo, 90133 Palermo, Italy; pietro.messina01@unipa.it

**Keywords:** deciduous molars, dental pulp, endodontics, indirect pulp therapy, pulpotomy

## Abstract

There is a paucity of information concerning vital pulp treatment outcomes in the undergraduate teaching setting. This study aimed to determine which type of deciduous molar, arch location, type of vital pulp therapy, and the number of carious surfaces involved had a better prognosis when carried out by undergraduate dental students. The method used was the review of clinical records of 590 patients with 600 deciduous molars, that visited the outpatient undergraduate dental clinics for vital pulp therapy. Statistical analysis used to determine the associations of tooth type, arch location, treatment type, and the number of carious surfaces involved in successful outcomes was logistic regression analysis with significance set at *p* < 0.05. According to the regression analysis model results, there was a significant association based on tooth type (*p* < 0.05) and arch location (*p* = 0.003). In addition, there was a significant association based on the type of treatment performed (*p* = 0.036). However, there was no significant association in success rates based on the number of carious surfaces involved (*p* = 0.873). In conclusion, second deciduous molars and maxillary deciduous molars had a better overall prognosis, and indirect pulp therapy was revealed to be more highly associated with successful treatment outcomes in comparison to ferric sulfate pulpotomy in our setting.

## 1. Introduction

The major factors in the success of a vital pulp therapy (VPT) procedure are the adequate diagnosis of the pulp and surrounding peri-radicular status, pulpal vitality preservation, and adequate vascularization of the pulp tissue [1]. The rationale of VPT is to provide a state that enables the formation of a hard tissue barrier and recovery of remaining tissue while preserving the functionality, therefore ensuring that a vital tooth remains in the oral cavity [1].

Loss of deciduous molar(s) (DM) prematurely can result in problems such as malocclusion, aesthetic, phonetic, and jaw functioning/growth alterations. According to the age of the child, these problems could be transient or permanent [2].

Three treatment modalities for treating vital DM are conventionally used in pediatric dental clinics, namely indirect pulp therapy (IPT), direct pulp capping (DPC), and pulpotomy [3]. IPT can be defined as the procedure aiming to protect and maintain the vitality of the pulp despite the presence of deep dentinal caries, which, if removed completely, could result in a pulp exposure [3]. DPC can be considered as a wound dressing of the exposed vital pulpal tissue. It is associated with low success rates also due to the high incidence of internal resorption in DM. For this reason, it has limited usage in pediatric dentistry [4]. On the other hand, pulpotomy aims at treating carious, exposed pulps in symptom-free teeth. The treatment success depends upon the ability of the radicular pulpal tissue to heal following surgical removal of the infected coronal pulp [2]. Endodontic microbiology has remained a focus for dental practitioners from as early as the 1960s. Good dental care can provide accurate information and knowledge on this very vast subject [5].

There are several factors to be considered when comparing vital pulp therapy in deciduous teeth. Al Zayer et al. and Vij et al. found the success rate of IPT to be 95%, and it was more likely to succeed in second DM than first DM [3,6]. It has been observed that IPT has a lower cost, better exfoliation pattern, and higher long-term treatment success rate in treating reversible pulpitis than pulpotomy [7]. In terms of choice of pulpotomy agents, due to the deleterious effects of formocresol (FC), ferric sulfate (FS) has been recommended as a replacement [8]. Furthermore, to the best of our knowledge, there are no published or accessible articles/studies available discussing the association of VPT outcomes to factors, such as type of tooth, arch location, type of treatment given, and the number of carious surfaces involved in DM. In most previous comparative studies, IPT has shown a success rate of 90% or above at the end of a follow-up period [9,10,11], whereas pulpotomy has demonstrated success rates ranging between 80–90% [7,12]. In the previous literature, to the best of our knowledge, only two studies have compared IPT with ferric sulphate pulpotomy (FSP) based on their treatment outcomes, and only one reported a significant difference [9]. In contrast, the other reported a difference, but it was not significant [13].

Apart from intra-operative factors, the clinical setting may influence the success rates of various operative procedures [3]. Interestingly, there is scarce evidence assessing the efficacy of VPT and the potential clinical factors, such as tooth, arch, and treatment type along with the number of involved carious surfaces, in the undergraduate (UG) teaching setting. In the past 15 years, stem-cell therapy has achieved meticulous scientific and commercial interest. Given its plasticity and the ability to differentiate into multiple cell lines along with unlimited self-renewal, stem-cell therapy would be able to provide promising results even in pediatric VPT [14].

Thus, this study aimed to determine the clinical treatment outcomes of VPT and their associations with factors such as type of DM, arch location, type of treatment, and the number of carious tooth surfaces destroyed when UG dental students perform the treatment.

## 2. Materials and Methods

### 2.1. Ethical Approval and Study Sample

The study was conducted in accordance with the Declaration of Helsinki, and ethical approval was obtained from the Human Research Ethics Committee of Universiti Sains Malaysia (USM) for this study (USM/JEPeM/19060358). This was a retrospective, record-review study. Purposive sampling, which is also known as judgmental, selective, or subjective sampling, was used in this study. A study sample of 590 pediatric patient records (350 males, 240 females) with 600 VPT-treated DMs in the last five years (starting from 1 September 2013 until 2 May 2018) were collected from the Records Unit, Hospital USM.

### 2.2. Consent for Using Patient Data

A consent form stating that the patient’s data can be used for future research purposes was signed by guardians of all 590 patients undergoing treatment at the UG pediatric dental unit.

### 2.3. Data Extraction

**Figure 1:**
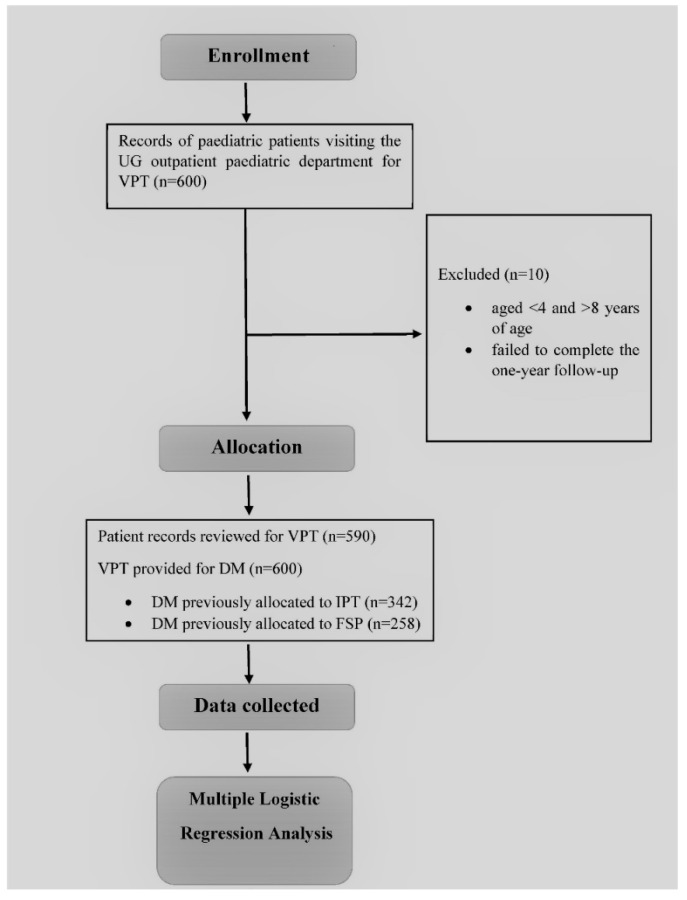
Flowchart of the study. Undergraduate (UG), Vital pulp therapy (VPT), Deciduous molar (DM), Indirect pulp therapy (IPT), Ferric sulfate pulpotomy (FSP).

Information, such as gender, age, type of treatment performed, materials used, final restoration material, type of DM, arch location, number of carious surfaces involved, treatment outcome, clinical follow-up evaluations after 1 week, 3 months, 6 months, and 12 months, and cause for treatment failure, were all extracted at the records unit on Microsoft excel spreadsheets (Microsoft^®^ Excel^®^ 2019 MSO 32-bit, Redmond, WA, USA) from the patient files who had undergone VPT in the UG pediatric dentistry unit at School of Dental Sciences, USM [9]. The summary of data collection and patient allocation for this study is shown in Figure 1.

### 2.4. Patient Recruitment/Allocation

**Figure 2:**
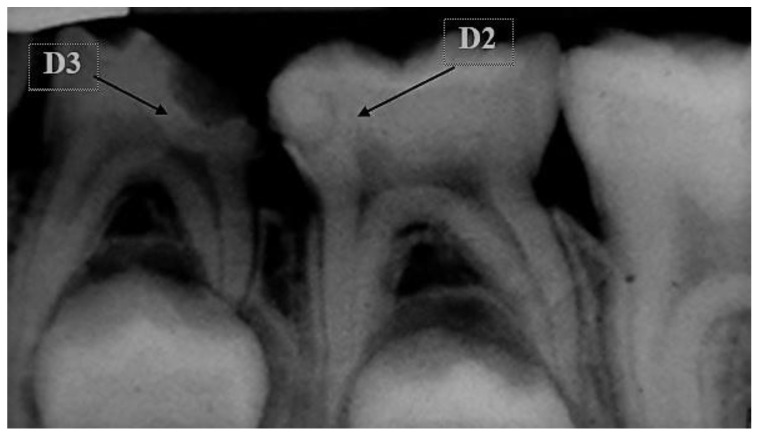
Preoperative periapical radiograph showing teeth 74 and 75. According to the American Dental Association Caries Classification System, tooth 74 showed disto-coronal radiolucency extending beyond the D3 level, i.e., into the pulp space and was treated with FSP. Tooth 75 shows mesio-coronal radiolucency extending beyond level D2, i.e., near the pulp horn and treated with IPT.

A comprehensive clinical consultation to assess the carious DM was carried out by UG students. Then, the radiographic examination was undertaken, which included bilateral bitewings and periapical radiographs to assess the caries extension on the teeth in question. A discussion was carried out between the UG student and a pediatric dental-specialist tutor to determine the type of VPT that was more indicated for the DM. If the clinical signs and symptoms conferred with a reversible pulpal injury, and no periapical or periradicular radiolucency was present radiographically, the tooth was allocated for either an IPT or an FSP [9]. Furthermore, the depth of caries was analyzed radiographically to choose between an IPT or FSP, following the American Dental Association Caries Classification System [15], as illustrated in Figure 2. Therefore, if the radiolucency was at or beyond a D2 level, the patient was allocated for an IPT, and if the radiolucency extended to a D3 level and involved the pulp chamber, the patient was allocated for an FSP.

### 2.5. Inclusion/Exclusion Criteria

All those pediatric patient records of patients aged between 4 to 8 years, in which DM presented with reversible pulpitis treated by an IPT/FSP and managed by UG students, were included.

DM that had naturally exfoliated shortly (3 to 6 months) after VPT were excluded. DM with signs and symptoms of irreversible pulpitis that required either pulpectomy or extraction were excluded. Patient records of children that failed to show up for the entire one-year duration of follow-up were excluded. Finally, patients who lacked pre-and post-treatment clinical records and pre-treatment radiographs were also excluded [9].

### 2.6. Operational Definitions

- Successful treatment—a treatment outcome was labeled as successful when there were no clinical signs of tenderness to percussion, normal tooth mobility, no sinus tract or abscess formation around the treated tooth, and no symptoms of spontaneous/nocturnal pain within a year (follow-up after 1 week, 3 months, 6 months, and 12 months) [16].
- Failed treatment—a treatment outcome was labeled as failed when any one of the following clinical or radiographic signs of failure were present, such as tenderness on percussion, abnormal tooth mobility according to Miller’s classification [17], presence of sinus tract or an abscess around the treated tooth, along with radiographic evidence of PDL widening, radiolucencies, or symptoms of spontaneous/nocturnal pain within a year (follow-up after 1 week, 3 months, 6 months, and 12 months) [16].

### 2.7. Procedure

Both IPT and FSP were performed by UG dental students from years 4 and 5 under strict supervision. Local anesthesia using mepivacaine hydrochloride (Scandonest 2%) was given before rubber dam application. For an IPT, caries were removed, and the cavity was rinsed with water and dried with air, using a triple syringe or a cotton pellet. A thin layer of calcium hydroxide (CH) was applied to the areas with limited remaining dentin. The remaining cavity was restored with a glass ionomer cement (GIC). After a one-week follow-up, a stainless-steel crown (SSC) was cemented as a permanent restoration using GIC luting cement.

For FSP cases, after adequate isolation with a rubber dam, caries were removed, and the pulp chamber was de-roofed to remove the coronal pulp. Coronal pulpal remnants were removed using a spoon excavator. Digital pressure was applied over the pulpal stumps using a saline-soaked cotton ball, and the cavity was cleaned. Once hemostasis was achieved, cotton soaked in 15.5% FS solution was placed over the pulpal stumps for 15 s, as per the manufacturers’ instructions, after being squeezed with gauze. Next, a layer of zinc oxide eugenol (ZOE)-based cement was applied, and the remaining cavity was filled with GIC. ZOE cement with low levels of eugenol was used; it has anti-inflammatory and local anesthetic properties in the dental pulp. Thus, while the application of ZOE temporary filling may aid in pulpal healing, on the other hand, high eugenol concentrations are cytotoxic [18]. After a one-week follow-up, if no signs or symptoms of pulpitis were seen, an SSC was cemented as a permanent restoration.

### 2.8. Statistical Analysis

The sample size was 600 DM, which was calculated using the two-proportion formula. Statistical analysis, including descriptive statistics, such as the frequency, mean, and standard deviation, was calculated using the SPSS version 24.0 software for Windows 10 (IBM SPSS Statistics, Armonk, NY, USA). Binary logistic regression analysis was carried out for data analysis, with the level of significance set at *p* < 0.05. Odds ratio (OR) and 95% confidence interval (CI) were used to identify the relationship between the clinical treatment outcomes of VPT performed on DM and associated factors.

## 3. Results

Table 1 shows the distribution of treatment outcomes of VPT according to the demographics and other variables, such as DM type, arch location, treatment type, and the number of carious surfaces involved. Six hundred records were assessed for data collection. 10 patients with 14 DMs failed to complete the entire follow-up period of one year. Records of ten patients were excluded, leaving behind records relative to 590 patients with 600 treated DM.

The association of the dependent variable (treatment outcomes) with the clinically observed independent variables (DM type, arch location, treatment type, and surfaces involved) was analyzed using logistic regression analysis. The initial univariate logistic regression model for all variables associated with treatment outcomes of VPT is shown in Table 2. The method used was “Enter” to evaluate the findings. It can be seen that the last variable, surfaces involved, has an insignificant relation with treatment outcomes.

**Figure 3:**
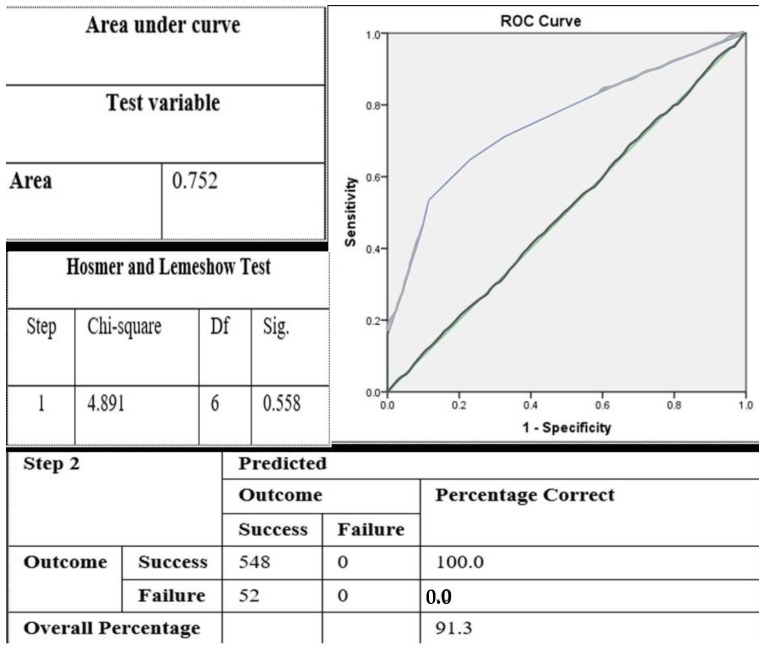
Assessment of goodness of fit of the final logistic regression model.

The final established model for variables significantly associated with treatment outcomes of VPT is shown in Table 3. Table 3 shows the final logistic regression model comparing the crude and adjusted OR and the elimination of the insignificant variable. Figure 3 shows the goodness of fit of the final model, which justifies the credibility of our model by fulfilling three necessary assumptions (namely the Hosmer–Lemeshow ratio, the classification table, and the area under the ROC curve). The insignificant variables were eliminated. According to the variable type of DM, with second DM as a reference, the OR was almost four times that of the first DM. According to arch type (i.e., maxilla vs. mandible), with maxillary arch as the reference, the OR was almost three times that of the mandibular arch. For type of treatment, with IPT as a reference, the odds ratio (OR: (95% CI)) was approximately twice that of FSP. Lastly, according to surfaces involved, there was no association between the number of surfaces involved and treatment outcome, so this variable was eliminated.

## 4. Discussion

The main finding of the present study is that IPT should be aimed for as the treatment of choice for reversible pulpal injuries when possible. Further analyses found that second DM and maxillary DM had a significantly higher rate of successful treatment outcomes, whereas the number of carious surfaces involved had no effect.

Treatments were performed by year four and five UG dental students. UG students of USM undergo vigorous clinical training starting from year three. It should be highlighted that all of the operators in this study were appropriately trained UG dental students who were very efficient on how to conduct the two types of VPT as well as how to offer an SSC as a permanent restoration of a DM following VPT. A study conducted in Brazil stated that the UG students performing partial caries removal and placing permanent restorations were, at all times, under strict supervision, similar to the scenario of our study [19]. A study that compared IPT procedures carried out by UG dental students and pediatric dental residents showed no significant differences, reporting that the operator experience did not influence the success of treatment outcomes since both were under the strict supervision of the pediatric dental specialists [3].

The second DM has larger pulp chambers as compared to the first DM. Additionally, the first DM commonly erupts before the second DM, making the former more prone and likely to become affected by caries. The significant difference between the first and second DM could be attributed to the following influencing facts: the cavity size, the root anatomy, restorability, and overall size of the DM [3].

The logistic regression analysis showed second DM to have four times higher successful treatment outcomes than the first DM. It can be speculated that a higher success rate with second DM is because they have larger-sized pulp chambers and potentially more progenitor cells.

Further possibilities would be that second DM erupt later than the first DM in a child’s life, making the latter more prone to the mastication and chemical insults associated with dietary intake. A study in agreement with our findings reported an 83% success rate in second DM compared to 61% in first DM that had undergone FC pulpotomy and 98% success rate of second DM versus 92% in first DM that had undergone IPT [6]. Another study in line with our study reported a higher success rate in second DMs that were treated with IPT compared to first DMs, with significant differences. The first DM was 4.4 (risk ratio) times more likely to fail than the second DM [3]. In a study contradicting our results, it was reported that treatment in second DM failed more often compared to first DM but with no significant differences [11]. The authors suggested that this was due to the insults and events acting over the restorations after a long-term function in the oral cavity. In addition, the cavity sizes of the second DM were larger when compared to the first DM, which needed more material for restoration [11]. These previous studies were either randomized controlled trials or prospective studies that usually have a high level of scientific evidence, though most of these either had a smaller sample size or a higher dropout rate compared to the current study.

The difference in findings when comparing maxillary and mandibular teeth can be explained as the maxillary bone has a greater amount of spongy bone and a thinner layer of cortical bone, richer in blood supply than the mandibular bone. According to logistic regression analysis, the odds of having clinically successful treatment outcomes after a VPT was almost thrice higher in the maxillary arch than the mandibular arch. In previous studies, no difference was reported while comparing maxillary and mandibular DMs [3,20]. Both these studies were unclear regarding the statistical values of the reported differences. The maxillary DM has three roots and a larger pulp chamber in comparison to mandibular ones [21]. Thus, it can be speculated that the reasons for a higher success rate in maxillary DMs are a more prosperous blood supply due to a larger pulp chamber and more than two roots for anchorage and as the source for vascular supply together with likely easier isolation in comparison to the mandibular DM.

Several studies have been carried using various materials for IPT or pulpotomy on DMs, which have been put forth to help general dental practitioners and specialists support their decision-making process [9,10,12,20,22,23]. Since both IPT and pulpotomy have identical indications [7], following the concepts of minimally invasive dentistry, it is speculated that IPT has better chances of preserving sound tooth structure than FSP.

Our results associating clinical treatment outcomes of IPT and FSP demonstrated a significant association (*p* = 0.036), indicating that IPT had a higher success rate (93.6%) clinically than FSP (88.4%). According to logistic regression analysis, the odds of successfully treating reversible pulpitis on a DM were almost twice better using IPT than FSP (odds ratio (OR) = 1.89 (1.04, 3.43; confidence interval (CI) = 95%)). Our results are in agreement with the previous retrospective studies and clinical trials reporting a clinical success rate above 90% with IPT [3,9,10,12,18,21,22,23,24,25] and an above 80% with FSP [7,8,9,12,26,27,28].

A survey conducted in Taiwan that included a year of follow-up also claimed that IPT had a higher success rate than FSP, but the difference was not significant [13]. Recently, a retrospective study compared the long-term (four-year follow-up) treatment outcomes of FSP with IPT in DM. It reported IPT to have significantly better outcomes than FSP [9]. While the results of these previous retrospective studies were from children treated under general anesthesia, all our cases were treated in the outpatient clinics. Unlike general anesthesia, which provides optimal conditions for treating uncooperative/disabled children [13], it is more stressful and difficult to manage children in outpatient clinics.

Comparative retrospective studies on DMs undergoing IPT and pulpotomy procedures also reported IPT to be superior in terms of treatment outcomes irrespective of the material used [9,29]. Pulpotomy procedures tend to increase the risk of displacing potentially infected dentin chips deeper into the healthy radicular pulp, which in turn also hinders the repair capacity of the radicular pulp [30]. We speculate that this could be one reason for the lower success rate in the current study.

When excavating deep caries, the number of tooth surfaces involved is either one or several. In this study, there was no significant association in clinical treatment outcomes between the two types of VPT based on the number of carious tooth surfaces involved. A study in agreement with the present revealed that the number of tooth surfaces involved had no association with the final treatment outcome [19]. In another study that evaluated IPT over a four-year follow-up period, no significant difference was found when comparing the number of surfaces involved (occlusal vs occluso-proximal) [11]. An expected difference could arise based on the access cavity design, but further research would be required for validation [31]. In the present study, all DM were restored with a SSC, which could be possible for no relation to surfaces involved with treatment outcomes. The results of this study are in line with previously reported literature showing that one of the reasons for a dramatically high success rate of IPT and pulpotomy is the use of an SSC as a permanent restoration [3,29,32].

Lastly, in the present study, successful VPT cases were assessed clinically only, following the ALARA guidelines [33], that is, to minimize radiation exposure at all times, especially in children, since they are at more risk to the stochastic biological effects of radiation [33]. Secondly, a DM has to exfoliate and be replaced by its permanent successor eventually. Thus, as long as it is not causing any harm to the daily masticatory functions or growth of the jaws or the permanent tooth, it should be left in place.

The limitations of this study were that post-VPT radiographs were not available for all the cases, and the follow-up period was only one year long since the UG students performing treatments had to complete the cases before their graduation. However, it can be noted from the results in Table 4 that all VPT cases in this study failed within the first three months. Methodological biases were due to the sampling method used in the present retrospective study, which was overcome during the analysis.

The present study differed from previous ones as an extensive sample was analyzed, providing impactful and effectual results. Overall, our findings suggest that comparable, successful outcomes can be obtained irrespective of the level of operator expertise. Furthermore, since all the operators in the current study underwent standardized preclinical training, inter-operator variability was reduced. Finally, previous studies did not assess outcomes based on molar type, arch location, and the number of carious surfaces involved. The results from the present study reiterate the importance of early diagnosis and caries management in DM.

We recommend future clinical trials for assessing the relationship between clinical variables, such as DM type, arch location, and treatment type, with the successful treatment outcomes through the findings of this study. Furthermore, future studies can evaluate the relationship between the depth of carious lesion and treatment outcomes of VPT.

## 5. Conclusions

Within the limitations of this study, it can be concluded that, based on the molar type and arch, second DM and maxillary DM had a significantly higher rate of successful treatment outcomes. IPT has a better clinical prognosis and a higher rate of successful treatment outcome than FSP performed on DM over a follow-up period of one year. The number of carious surfaces involved did not influence treatment outcomes in DM. Clinical treatment outcomes of VPT in a UG teaching clinic are comparable to those previously reported in alternative settings.

## Figures and Tables

**Table 1:** Distribution of different variables based on the type of VPT performed.

	Based on Sex and Age		
	Frequency	Percentage (%)	
**Gender**			
**Male**	350	59.3	
**Female**	240	40.7	
**Total**	590	100.0	
**Age**			
**Mean (SD)**	6.54 (1.18)		
	**Based on Deciduous Molar Type**		
**Treatment Outcome**	**S (%)**	**F (%)**	**Total**
**1st Deciduous Molar**	237 (85.3)	41 (14.7)	278
**2nd Deciduous Molar**	311 (96.6)	11 (3.4)	322
	**Based on Arch Type**		
**Treatment Outcome**	**S (%)**	**F (%)**	**Total**
**Maxillary Arch**	226 (96.6)	8 (3.4)	234
**Mandibular Arch**	322 (88.0)	44 (12.0)	366
	**Based on treatment type**		
**Treatment Outcome**	**S (%)**	**F (%)**	**Total**
**Type of VPT**			
**IPT**	320 (93.6)	22 (6.4)	342
**FSP**	228 (88.4)	30 (11.6)	258
**Final VPT Outcome**	548 (91.3)	52 (8.6)	600
	**Based on Number of Surfaces Involved**		
**Treatment Outcome**	S (%)	F (%)	Total
**One surface involved**	331 (91.2)	32 (8.8)	363
**Multiple surfaces involved**	217 (91.6)	20 (8.4)	237

SD-Standard deviation; S-Successful outcomes; F-Failed outcomes.

**Table 2:** Univariate logistic regression model showing the odds ratio (OR) (95% confidence interval (CI)) for successful treatment outcomes according to all clinically observed variables.

	B	S.E.	Wald	*p*-Value	OR
**Type of Deciduous Molar** **(2nd D.M. as an indicator)**	1.587	0.353	20.534	<0.0001 *	4.89 (2.46–9.72)
**Arch Type** **(Maxillary as indicator)**	1.352	0.348	11.752	0.001 *	3.86 (1.78–8.36)
**Treatment Type** **(IPT as indicator)**	0.652	0.294	4.881	0.027 *	1.91 (1.08–3.40)
**Surfaces Involved** **(One surface as indicator)**	0.048	0.298	0.026	0.873	0.95 (0.53–1.71)

* Significant *p*-value.

**Table 3:** Logistic regression model showing the crude and adjusted OR (95%CI) for successful treatment outcomes according to clinically observed variables. * Significant *p*-value.

Variables	B	S.E.	Wald	Crude OR	*p*-Value	Adjusted OR	*p*-Value
**Type of Deciduous Molar** **(Second DM)**	1.434	0.355	16.331	4.89 (2.46, 9.72)	<0.001 *	4.19 (2.09, 8.41)	<0.001 *
**Arch Type (Maxillary)**	1.183	0.401	8.702	3.86 (1.78, 8.35)	0.001 *	3.27 (1.49, 7.17)	0.003 *
**Type of Tx** **(IPT)**	0.634	0.303	4.379	1.91 (1.08, 3.40)	0.027 *	1.89 (1.04, 3.42)	0.036 *

**Table 4:** Distribution of the clinical sequelae of failed cases of DM based on the clinical manifestation of failure and duration.

Follow-Up Period		1 Week	3 Months	6 Months	12 Months
Cause of Failure/Total (n)		IPT/FSP	IPT/FSP	IPT/FSP	IPT/FSP
**Nocturnal pain**	27	16/9	0/2	-	-
**Grade II/III Mobility**	10	-	1/9	-	-
**Sinus tract**	5	-	2/3	-	-
**Abscess**	10	1/2	2/5	-	-
**Total**	52	17/11	5/19		
		28	24		

## Data Availability

The data used to support the findings of this study are available from the corresponding author upon request.
